# Slope position influences kiwifruit (*Actinidia chinensis* Planch.) yield through soil water distribution, water consumption, and growth physiology in a subtropical humid region of China

**DOI:** 10.3389/fpls.2026.1847750

**Published:** 2026-05-29

**Authors:** Lin Zhu, Zhiyu Chen, Wei Wang, Jintao Xu, Yiwen Qiu, Shuai Tan

**Affiliations:** 1 Yunnan Key Laboratory of Efficient Utilization of Agricultural Water Resources and Intelligent Control, Faculty of Modern Agricultural Engineering, Kunming University of Science and Technology, Kunming, China; 2 Yunnan International Joint Laboratory of Intelligent Agricultural Engineering Technology and Equipment, Faculty of Modern Agricultural Engineering, Kunming University of Science and Technology, Kunming, China; 3 College of Water Resources & Civil Engineering, Hunan Agricultural University, Changsha, China; 4 Seasonal Arid Region, Water-Soil-Crop System Observation and Research Station of Yunnan Province, Faculty of Modern Agricultural Engineering, Kunming University of Science and Technology, Kunming, China

**Keywords:** growth physiology, kiwifruit, sloping cultivation, soil water, yield

## Abstract

**Introduction:**

Yield formation of kiwifruit (*Actinidia chinensis* Planch.) is associated with soil water conditions. In the hilly areas of subtropical humid regions in China, sloping cultivation is a common practice. Slope position influences soil water distribution, possibly inducing variations in kiwifruit growth, physiology, and yield. However, the underlying pathways through which sloping cultivation affects kiwifruit yield remain unclear.

**Methods:**

Therefore, a two-year field investigation was conducted to evaluate the differences in soil water content (SWC), actual evapotranspiration (ET_c act_), growth physiology, and yield of kiwifruit between upper and lower slopes. The primary factors influencing kiwifruit yield were identified using the random forest and partial least squares path modeling (PLS-PM).

**Results:**

Results showed that SWC on lower slopes was significantly higher than that on upper slopes, exceeding the appropriate SWC for kiwifruit growing and yield formation. In contrast, upper slopes exhibited superior growth physiology performance, yield, and water productivity (WP_c_), compared to lower slopes. Specifically, upper slopes significantly increased ET_c act_, WP_c_, chlorophyll content index (CCI), shoot dry weight (SDW), fruit number (FN), and yield by 4.05%, 34.8%, 10.3%, 52.8%, 33.8%, and 39.3%, respectively. PLS-PM revealed that yield was directly influenced by FN and fruit weight. Soil water (average SWC and SWC at fruit development stage) and water consumption (ET_c act_ and ET_c act_ at fruit development stage) indirectly and positively affected yield by influencing growth physiology (CCI and leaf dry weight per unit shoot leaf area) and yield components. These pathways collectively explained 93.1% of yield variations.

**Discussion:**

This study can provide a reference for kiwifruit water management under sloping cultivation in subtropical humid regions. Specifically, supplement irrigation on upper slopes is recommended during dry periods, while drainage improvement is recommended on lower slopes to avoid excessive soil water accumulation or prolonged high SWC.

## 1 Introduction

Kiwifruit (*Actinidia chinensis* Planch.), an important economic crop in subtropical regions, is abundant in trace elements and essential vitamins, earning the reputation of the “King of Fruits” (Xue et al., 2019). It thrives in sunlight but is sensitive to intense direct exposure, and its fleshy root system is highly susceptible to soil water fluctuations (Calabritto et al., 2024; Zheng et al., 2024). In subtropical humid climates, frequent short-duration heavy rainfall during the rainy seasons often causes excessive water accumulation in the root zone. Notably, precipitation is mostly concentrated at the fruiting stage of kiwifruit (from May to September), adversely affecting yield formation, increasing disease risk, and severely limiting local production. Cultivation on sloping land can facilitate drainage during the rainy seasons, consequently reducing soil erosion and excessive water accumulation (Monsieurs et al., 2015). Additionally, this practice can provide suitable light and soil conditions to support root respiration, promoting photosynthetic efficiency, and nutrient accumulation (Yao et al., 2024), ultimately contributing to higher yield. Considering these advantages, sloping land cultivation is widely used for sustainable kiwifruit production in subtropical humid regions of China.

Slope position, as a critical topographic factor, poses an indirect effect on crop growth physiology by regulating spatial and temporal soil water distribution, thus influencing yield formation (Zhu et al., 2015). Driven by gravity, soil water moves from upper to lower slopes, typically resulting in higher soil water storage on lower slopes (Shan et al., 2023), which in turn leads to differences in crop water consumption at different slope positions (Zhu et al., 2015; Horel and Zsigmond, 2023). In heavy rainfall regions, soil on lower slopes is more prone to reaching saturation, consequently limiting further infiltration due to its higher initial soil water content (SWC) (Chen et al., 2021; Phi et al., 2013). Tang et al. (2014) found that SWC on lower slopes were 50.9%-77.3% higher than that on upper slopes under the same planting pattern in hilly dryland of southern China. Soil water status impacts crop nutrient uptake, root development dynamics, and crop adaptability to soil conditions. Both water excess and deficit possibly cause poor fruit development and a low fruit-setting rate in kiwifruit, severely constraining yield formation. In general, appropriate soil water condition for kiwifruit growth and yield ranges from 60% to 80% of field capacity (FC) (He et al., 2023). For instance, Zheng et al. (2023) demonstrated that kiwifruit subjected to water stress (about 15%−45% of full irrigation amount) during the fruit expansion stage, exhibited severe reductions in yield.

Crop growth and physiological processes are closely associated with yield formation. Previous studies have pointed out that crop yield and its components correlated positively with leaf number (LN), leaf area, shoot length (SL), and leaf dry weight (LDW) (Rana et al., 2023). Besides, leaf morphology (e.g. leaf area and rounder leaves) had a strong positive impact on fruit sugar content (Rowland et al., 2020). Notably, lower slopes typically exhibit significantly more leaves compared to middle-upper slopes due to higher SWC (Zhang et al., 2019), potentially increasing photosynthetic capacity for dry matter accumulation (Heslop et al., 2023; Li et al., 2025) In addition, chlorophyll content is another key factor in yield formation and is highly sensitive to soil water availability (Yetik and Candoğan, 2023; Ghimire et al., 2024). Slope position indirectly affects chlorophyll content by altering soil water distribution, thereby influencing yield. Generally, higher chlorophyll content is observed on lower slopes, contributing to higher crop yield due to higher SWC and nutrient availability (Chen et al., 2023; Koide and Kobayashi, 2025). Considering the uneven precipitation in subtropical humid regions of China, although lower slopes typically maintain higher SWC than upper slopes, the soil water levels may exceed the appropriate range for kiwifruit development, potentially creating unfavorable growth conditions. Conversely, possibly moderate stress on upper slopes may enhance yield of kiwifruit. Therefore, it is necessary to clarify the direct and indirect effects of slope position on kiwifruit growth and physiology for optimizing yield in subtropical humid regions.

Sloping position can cause non-uniform distribution of soil water, which in turn leads to variations in crop water consumption and growth physiology. These differences ultimately regulate yield performance through both direct and indirect pathways. However, the primary determinants of kiwifruit yield remain insufficiently elucidated under sloping cultivation in subtropical humid regions. Based on this, a field investigation was conducted in a subtropical humid region to systematically evaluate the effects of slope position on kiwifruit yield. The objectives of this study were: (1) to compare the differences in SWC, water consumption, growth physiology, and yield between upper and lower slopes; (2) to identify the potential factors influencing yield; and (3) to elucidate the pathways through which key factors influenced yield under sloping cultivation. This study can provide a reference for water management in kiwifruit cultivation on sloping land in subtropical humid regions.

## 2 Materials and methods

### 2.1 Experimental site description

**Figure 1:**
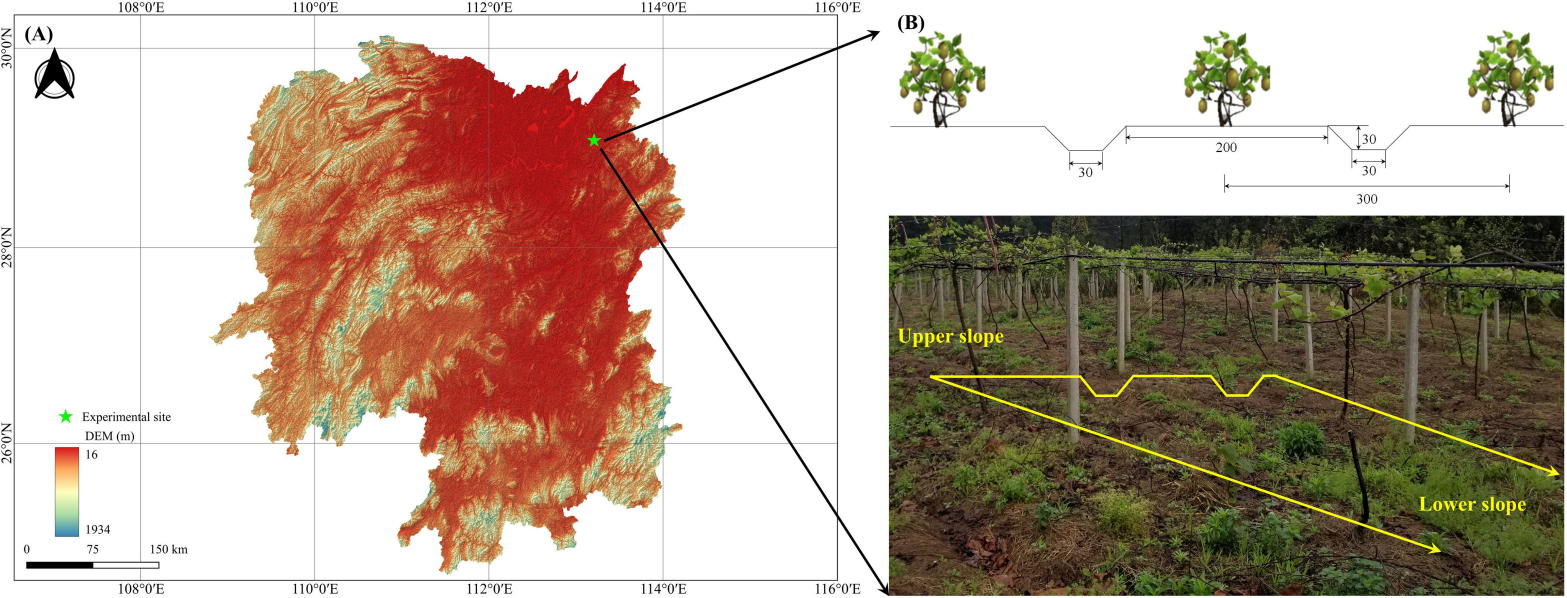
The location of the experimental site **(A)** and kiwifruit plant arrangement **(B)**.

A field investigation was conducted from March to August in both 2019 and 2020 in Gangkou Town, Yueyang County, Hunan Province, China (29°04’46’’ N, 113°10’24’’ E, mean altitude of 60.0 m, **Figure 1A**). The site features hilly terrain with typical slopes ranging from 10° to 15°. The area has a subtropical humid monsoon climate, characterized by distinct seasonal precipitation, with an annual average of 1423 mm. The mean annual temperature is 16.9 °C, the mean wind speed is 2.4 m s^−1^. The annual sunshine duration reaches approximately 1656 h, and the frost-free period lasts about 270 days. The soil at the experimental site is classified as silty clay loam (12.3% sand, 53.8% silt, and 33.9% clay) according to USDA soil texture classification system. In the 0−120 cm soil profile, the average soil bulk density, FC, and saturated soil water content (*θ_s_*) were 1.40 g cm^−3^, 0.297 cm^3^ cm^−3^, and 0.448 cm^3^ cm^−3^, respectively, with a pH of 4.50 and an average organic matter content of 11.0 g kg^−1^. Basic physical properties of the 0−120 cm soil profile are detailed in **Table 1**. The soil water characteristic curve for the 0−120 cm profile was determined using the centrifuge method to estimate soil hydraulic parameters based on the van Genuchten model (van Genuchten, 1980). The residual SWC (*θ_r_*) was 0.0755 cm^3^ cm^−3^, the inverse of the air-entry value (*α*) was 0.0173 cm^−1^, the empirical coefficient (*n*) was 1.339, and the soil saturated hydraulic conductivity (*K_s_*) was 17.92 cm d^−1^.

**Table 1:** Soil physical properties of the 0–120 cm profile in the experimental site.

Soil depth (cm)	Bulk density (g cm^−3^)	*θ_s_* (cm^3^ cm^−3^)	FC (cm^3^ cm^−3^)
0−5	1.43±0.04	0.484±0.026	0.303±0.007
>5−10	1.25±0.05	0.407±0.009	0.260±0.014
>10−20	1.28±0.03	0.448±0.023	0.304±0.014
>20−30	1.22±0.04	0.480±0.014	0.304±0.013
>30−40	1.31±0.04	0.464±0.011	0.295±0.008
>40−60	1.51±0.05	0.456±0.034	0.317±0.007
>60−80	1.57±0.02	0.447±0.017	0.300±0.007
>80−100	1.49±0.04	0.425±0.019	0.291±0.013
>100−120	1.54±0.05	0.423±0.010	0.304±0.008
0−80	1.41±0.04	0.455±0.021	0.302±0.009
0−120	1.45±0.04	0.445±0.019	0.301±0.010

*θ_s_* and FC represent saturated water content and field capacity, respectively.

### 2.2 Kiwifruit field management

In 2016, two-year-old kiwifruit vines (*Actinidia chinensis* cv. ‘Hongyang’) were planted on sunny slopes in the orchard. The vines were 5−6 years old during the investigation period (2019−2020) and had reached full bearing maturity. According to the technical regulations of China for kiwifruit production in hills or mountains (NY/T 5108-2002), the orchard was divided into multiple operating units of 0.3−0.5 ha. The width and length of each operating unit were 40−50 m and 80−85 m, respectively. The elevation difference between the top and bottom of each unit ranged from 12 m to 20 m. The pergola system was adopted for cultivation, with vines planted on ridges at a planting spacing of 2.0 m and row spacing of 3.0 m (**Figure 1B**). There were drainage ditches with a depth and bottom width of 30 cm between the planting rows. To ensure pollination, a 10:1 female-to-male ratio (10 female vines per male vine) was used. Excluding male vines, the planting density was 1515 vines ha^−1^. The phenological cycle of the vines commenced with bud-leaf development in early March and ended with harvest in mid-to-late August. As the deep groundwater depth (> 20 m) and abundant precipitation during the growing season, the water supply for the vines solely depended on precipitation. Weeds exceeding 20 cm in height were removed mechanically, and pruning was performed twice annually (spring and winter). Organic fertilizers were applied as both base and topdressing at a rate of 75 t ha^−1^ to maintain soil fertility.

### 2.3 Kiwifruit field investigation

Given the small area (40−50 m × 80−85 m) and low elevation difference (12−20 m) within each operating unit of kiwifruit, multi-point sampling was carried out at two representative slope positions (upper and lower) during two growing seasons to evaluate the effects of slope position on soil water distribution and water consumption. Eight sampling points were arranged at each slope position in a representative operating unit, with a minimum spacing of 15 m between points to ensure spatial independence. At each sampling point, three measurement replicates were taken to ensure statistical reliability. Field data included meteorological data, SWC, growth physiology indicators, as well as yield and its components.

### 2.4 Data collections and calculations

#### 2.4.1 Meteorological data collection

**Figure 2:**
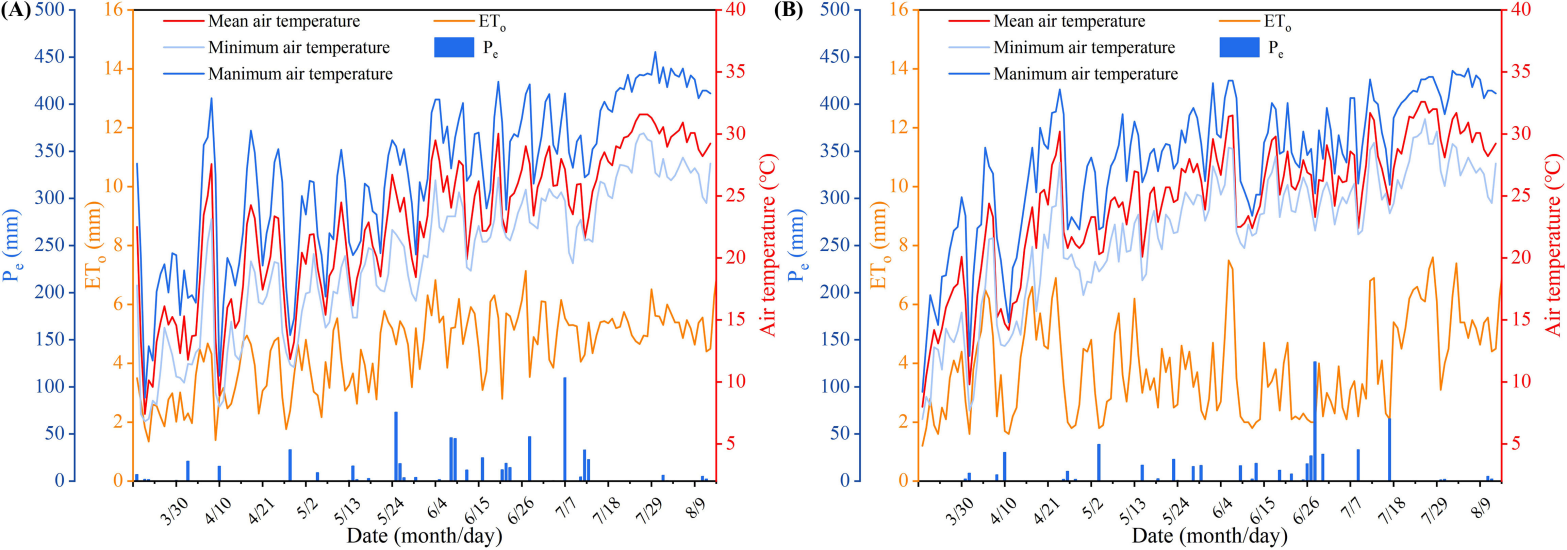
ET_o_, air temperature, and effective precipitation (P_e_) in the 2019 **(A)** and 2020 **(B)** growing seasons of kiwifruit.

A HOBO U30 automatic weather station (Onset, Bourne, MA, USA) was installed at a distance of approximately 30 m from the experimental site to collect hourly meteorological data during the growing season, including precipitation, solar radiation, maximum and minimum temperatures, relative humidity, and wind speed at a height of 2 m. Daily reference evapotranspiration (ET_o_) was calculated using the Penman-Monteith equation (Allen et al., 1998). Both ET_o_ and effective precipitation illustrated pronounced temporal fluctuations (**Figure 2**). ET_o_ ranged from 1.3 to 7.14 mm for the 2019 season and 1.0 to 7.6 mm for the 2020 season, with higher values observed during summer (June to August). A total of 36 and 32 effective precipitation events occurred during the two growing seasons, predominantly in July, accumulating 617.5 mm in the 2019 season and 598.6 mm in the 2020 season, respectively. It can be seen that the meteorological conditions during both seasons were similar.

#### 2.4.2 Soil water content measurements and calculations

Time domain reflectometry (TDR, IMKO Micromodultechnik GmbH, Bodenheim, Germany) was used to determine SWC (cm^3^ cm^−3^) during the growing season. There was a significant linear relationship between actual SWC determined by the oven-drying method (SWC_act_) and measured SWC using TDR (SWC_act_ = SWC_TDR_ + 0.102, *r*^2^ = 0.746, *n* = 65, *p* < 0.001). TRIME tubes (150 cm length) were installed at distances of 0, 30, and 60 cm from the vine trunks, with 15 cm of each tube left above ground to prevent rainwater intrusion. SWC was measured at 20-cm depth intervals within the 0−120 cm soil profile, representing an average SWC for each soil layer. These measurements were conducted at the beginning of key growth stages of kiwifruit, i.e., bud-leaf development (G1, mid-March), flowering (G2, mid-May), fruit setting (G3, mid-June), fruit development (G4, early July), fruit maturity (G5, late July), and harvest (G6, mid-August).

The total actual evapotranspiration (ET_c act_) and ET_c act_ at each growth stage, reflecting water consumption of kiwifruit during the growing season, was determined using the water balance equation (Zhang et al., 2023):


$${\text{ET}}_{\text{c act}}={\text{P}}_{\text{e}}+\text{I}+\text{Q}+\text{Δ}\text{SWS}$$

$${\text{P}}_{\text{e}}=\{\begin{array}{ll} 0 & \text{P}<5\\ 0.75(\text{P}-5) & \text{P}\geq 5 \end{array}$$

Where P_e_ is the effective precipitation (mm); I is the irrigation amount (mm), which is set to zero due to rain-fed irrigation for kiwifruit in the experimental site; Q is the water exchange capacity in the main root zone (mm), with negative values indicating leakage and positive values indicating upward water supply from the lower to upper soil layers, calculated from water flux using Darcy’s law and parameters of van Genuchten model (van Genuchten, 1980; Mualem, 1976); and ΔSWS is the variation in water storage in the main root zone.

#### 2.4.3 Growth physiology measurements

At harvest, three kiwifruit vines with uniform growth and no signs of pests or diseases were selected at each sampling point. From each vine, five fully expanded green leaves on fruiting shoots within the middle canopy were collected from four directions (east, south, west, and north). Relative leaf chlorophyll content, expressed as the chlorophyll content index (CCI) (Parry et al., 2014; Padilla et al., 2018), was measured using a CCM-200 PLUS chlorophyll meter (Opti-Sciences Inc., New Hampshire, USA), with three replicate measurements per leaf. Additionally, four new shoots oriented in different directions were cut off at the base to determine leaf number (LN), shoot length (SL, cm), single leaf area (SLA, cm^2^), as well as shoot and leaf dry weights (SDW and LDW, g shoot^−1^). SL was measured from the base to the shoot tip using a tape with a precision of 1 mm. The maximum length and width of all green leaves per shoot were also measured with the tape to estimate SLA. Leaf area was calculated by multiplying maximum leaf length and width by a factor of 0.763 (Tan et al., 2018). This factor was derived from a linear regression (*R*^2^ = 0.994, *n* = 43, *p* < 0.001) validated against measurements obtained from an AM 300 leaf area meter (ADC BioScientific Ltd., UK). Shoots and leaves were separated, cleaned of surface dust, oven-dried at 105 °C for 1 h, then at 85 °C until a constant weight was achieved. To reduce the influence of variation in shoot and leaf dry weights, shoot dry weight per unit shoot length (SSL, g cm^−1^) and leaf dry weight per unit shoot leaf area (LLA, g m^−2^) were calculated as follows:


SLA=0.763·L·W

$$SSL=\frac{SDW}{SL}$$

$$LLA=\frac{10^{4}LDW}{\sum_{i=1}^{n}SLA}$$

Where *L* is the maximum leaf length (cm). *W* is the maximum leaf width (cm), and *n* is the leaf number of the selected shoot.

#### 2.4.4 Yield components

At harvest, three representative kiwifruit vines with consistent growth and free from diseases were selected at each sampling point to determine yield. After removing undersized or damaged fruits, the number of fruits per vine (FN) was counted. All fruits from each vine were harvested and weighed using an electronic balance with a precision of 0.01 g to determine individual fruit weight (FW) and yield per vine. Notably, yield per vine was directly obtained by weighing the total harvested fruits. The yield per vine was converted into unit area yield (t ha^−1^) based on the planting density (1515 female vines ha^−1^). Combining the yield (Y, kg ha^−1^) and ET_c act_, water productivity (WP_c_, kg m^−3^) can be calculated as follows (Fernández et al., 2020):


$$WP_{c}=\frac{100Y}{ET_{c\;act}}$$

### 2.5 Data processing and statistical analysis

Given the similar meteorological conditions in 2019 and 2020 growing seasons and no significant effects of year and the interactions between year and slope on the measurements (*p* > 0.05), the values for each sampling point were averaged across the two seasons for analysis. SPSS 21.0 (IBM Corp., Armonk, NY, USA) and Origin 2024 (OriginLab, Northampton, MA, USA) were used to analyze the effects of slope position on soil water and yield. Data following a normal distribution were analyzed using one-way analysis of variance (ANOVA), whereas non-parametric Mann-Whitney U test were applied for non-normally distributed data. A two-way ANOVA was also carried out to evaluate the effects of slope position, soil depth, and their interactions on SWC. When a significant difference (*p* < 0.05) was detected, multiple comparisons were carried out using the least significant difference (LSD) method, with different lowercase letters indicating significance at the *α* = 0.05 level.

Considering the non-normal distribution in some variables, Spearman correlation analysis and Mantel test were used to explore the relationships between yield performance and soil water conditions (average SWC and SWC at G1-G6 in main root zone), water consumption (ET_c act_ and ET_c act_ at G1-G5), and growth physiology (CCI, LN, SL, SLA, SDW, LDW, SSL, and LLA) indicator. Subsequently, the random forest (RF) method was applied to identify potential important factors influencing kiwifruit yield based on a 90% cumulative contribution threshold with ntree = 1500 and nrep = 300 permutation tests. This method can handle high-dimensional data processing and nonlinear interactions among variables (Niu et al., 2026). Variable importance was assessed by the increase in mean squared error. Multicollinearity among potential variables was assessed using variance inflation factor (VIF) (Gao et al., 2026). Variables with VIF > 5 were removed. Finally, partial least squares path modeling (PLS-PM) was conducted to determine the influence pathways and primary factors affecting kiwifruit yield. To further evaluate the stability and significance of the path coefficients, bootstrap resampling (*n* = 6000) was used. The Mantel test, RF, and PLS-PM were performed using the ‘vegan’, ‘randomForest’, ‘rfPermute’, ‘ggplot2’, and ‘plspm’ packages in R software.

## 3 Results

### 3.1 Effects of slope position on spatial and temporal soil water distribution

#### 3.1.1 Spatial distribution of soil water

**Figure 3:**
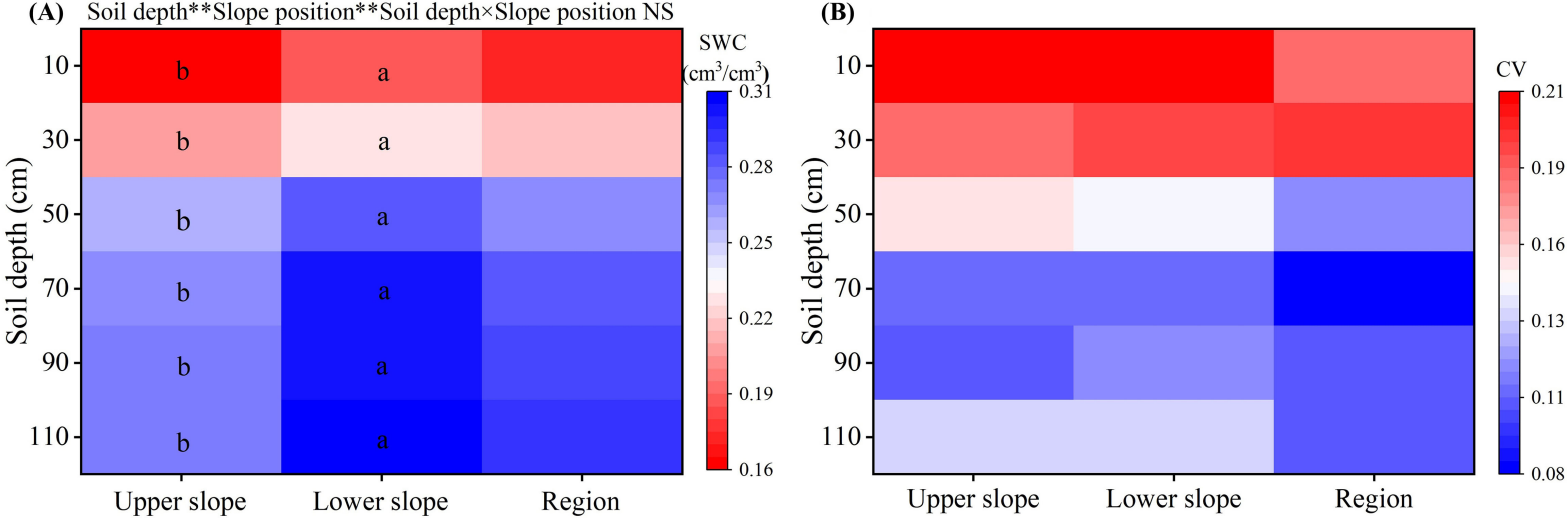
Soil water content distribution **(A)** and coefficient of variation **(B)** in the 0−120 cm soil profile during the growing season of kiwifruit. Different lowercase letters denote significant differences between upper and lower slopes in the same soil layer at *α* = 0.05. **p < 0.01; NS, no significant difference. Region values represent the average soil water content of upper and lower slopes. *n* = 144 for upper and lower slopes, and *n* = 288 for the region.

Both slope position and soil depth significantly influenced SWC (*p* < 0.01), but their interaction was not statistically significant (*p* > 0.05, **Figure 3**). During the growing season, SWC varied consistently with soil depth on both upper and lower slopes (**Figure 3A**). In the 0−80 cm soil layer, SWC generally increased with soil depth, exhibiting an increase of 58.4% to 63.6% from the 0−20 cm to the 60−80 cm soil layer. Below 80 cm, SWC stabilized at 0.272−0.275 cm^3^ cm^−3^ (90.5%−93.5% FC) on upper slopes and 0.304−0.310 cm^3^ cm^−3^ (approach to 100% FC) on lower slopes, demonstrating that the 0−80 cm soil layer can represent the main root water exchange zone (effective root zone) for kiwifruit water uptake. The coefficient of variation (CV) of SWC in the vertical profile during the growing season ranged from 0.079 to 0.213, indicating low and moderate variability (0.1 < CV < 1.0). In the main root zone (0−80 cm), the CV decreased with increasing depth (**Figure 3B**), revealing greater fluctuations in surface SWC and relative stability in deeper layers. Additionally, SWC in each layer was significantly lower on upper slopes compared to lower slopes (*p* < 0.05), with reductions of 8.96%−15.85%.

#### 3.1.2 Temporal distribution of soil water

**Figure 4:**
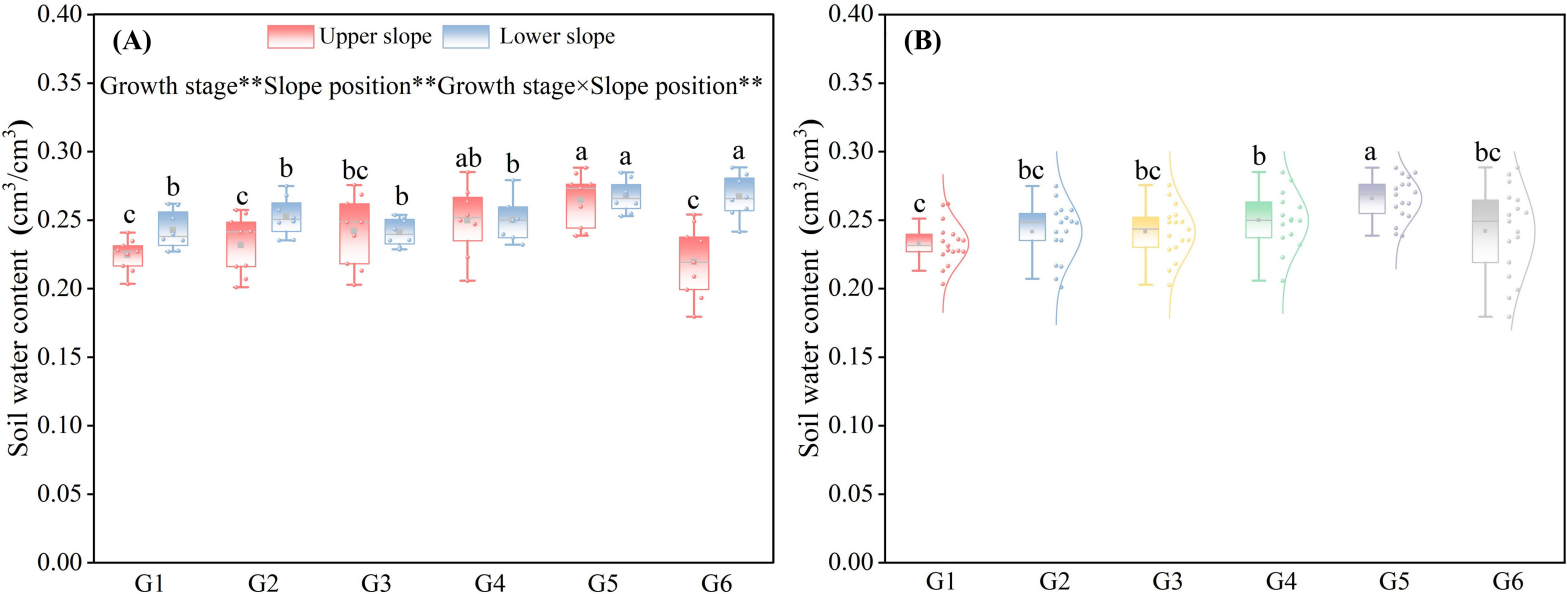
Variations in soil water content on upper and lower slopes **(A)** and in the region **(B)** in the main root zone (0−80 cm) of kiwifruit during the growing season. G1−G6 correspond to bud-leaf development, flowering, fruit setting, fruit development, fruit maturity, and harvest stages, respectively. Different lowercase letters denote significant differences among different growth stages at the same slope position at *α* = 0.05. **p < 0.01. *n* = 24 for upper and lower slopes, and *n* = 48 for the region at each growth stage. The region values are presented for reference only and were not used in statistical comparisons.

Consistent variations in SWC in the main root zone of kiwifruit were observed on both upper and lower slopes, with an initial increase followed by a decline (**Figure 4**). Growth stage, slope position, and their interaction significantly influenced SWC (*p* < 0.01). The maximum SWCs were detected (0.264−0.267 cm^3^ cm^−3^) at G5, which was significantly higher than SWCs at other growth stages. This increase is attributed to higher precipitation from July to August (**Figure 2**). Moreover, average SWC in the main root zone ranged from 0.219 to 0.264 cm^3^ cm^−3^ for upper slopes and from 0.241 to 0.267 cm^3^ cm^−3^ for lower slopes (**Figure 4B**), corresponding to 72.5%−87.4% FC and 80.1%−88.4% FC, respectively. Similar to the vertical profile of SWC, upper slopes exhibited significantly lower SWC in the main root zone in comparison to lower slopes during the growing season (*p* < 0.05), with reductions of 7.4%−18.0%, except at G3−G5 (**Figure 4A**).

### 3.2 Effect of slope position on ET_c act_ during kiwifruit growing season

According to the water balance equation, ET_c act_ during the growing season ranged from 560.2 to 617.2 mm (**Table 2**), with low variability at two slope positions and the study area (CV < 0.1). Specifically, ET_c act_ on upper slopes was significantly higher by 4.05% than that on lower slopes (*p* < 0.05).

**Table 2:** Descriptive statistics of ETc act during the growing season of kiwifruit.

Slope position	*n*	Maximum	Minimum	Mean	S.D.	CV
		-––mm-––				
Upper	24	617.2	579.0	600.8 a	15.3	0.025
Lower	24	605.8	560.2	577.4 b	13.0	0.022
Region	48	617.2	560.2	590.3	18.0	0.030
Analysis of variance (*p* value)						
Slope position				< 0.01**		
Year				0.155^NS^		
Slope position × Year				0.986^NS^		

Region includes both upper and lower slopes. S.D. indicates standard deviation. Different lowercase letters within a column denote significant differences between upper and lower slopes at *α* = 0.05. The region values are presented for reference only and were not used in statistical comparisons. **p < 0.01; NS, no significant difference.

To further characterize water consumption patterns of kiwifruit, ET_c act_ was analyzed at four key growth stages (**Table 3**). As flowering and fruit setting generally represent the transition from vegetative to reproductive growth, these stages were combined into a single flowering-fruiting stage (G2−3) for analysis. ET_c act_ initially increased and subsequently declined during the growing season, with the lowest proportion detected at G1 (4.3%−4.6%) and the highest proportion at G4 (77.8%−80.6%).

**Table 3:** Descriptive statistics of ETc act proportion at key growth stages of kiwifruit.

Slope position	*n*	ET_c act_ (mm)	Proportion of ET_c act_ (%)			
			G1	G2−3	G4	G5
Upper	24	600.8	4.6	11.6	77.8	6.0
Lower	24	577.4	4.3	10.4	80.6	4.7
Region	48	590.3	4.5	11.0	79.1	5.4

G1 is bud-leaf development stage; G2−3 is flowering-fruiting stage; G4 is fruit development stage; and G5 is fruit maturity stage.

### 3.3 Effects of slope position on growth physiology of kiwifruit

At harvest, CCI, LN, SL, SLA, SDW, LDW, SSL, and LLA were 21.4−23.6, 10.7−12.8, 50.4−68.0 cm, 77.0−82.9 cm^2^, 7.2−11.0 g shoot^−1^, 13.2−15.7 g shoot^−1^, 0.16−0.17 g cm^−1^, 150.5−161.5 g cm^−2^, respectively (**Table 4**). Slope position had a significant effect on CCI and SDW (*p* < 0.05). Compared with lower slopes, upper slopes significantly increased CCI by 10.3% and SDW by 52.8%, respectively. Other indicators of growth were slightly higher on upper slopes than on lower slopes (*p* > 0.05). These findings indicate that the upper slope position can promote kiwifruit growth, dry matter accumulation, and chlorophyll synthesis under the subtropical humid region.

**Table 4:** Statistics of growth physiology indicators of kiwifruit at harvest.

Slope position	*n*	CCI	LN	SL (cm)	SLA (cm^2^)	SDW (g shoot^−1^)	LDW (g shoot^−1^)	SSL (g cm^−1^)	LLA (g m^−2^)
Upper	24	23.6 ± 8.9a	12.8 ± 6.8a	68.0 ± 22.9a	82.9 ± 11.5a	11.0 ± 3.2a	15.7 ± 6.9a	0.17 ± 0.06a	161.5 ± 43.7a
Lower	24	21.4 ± 9.2b	10.7 ± 4.5a	50.4 ± 26.2a	77.0 ± 15.1a	7.2 ± 4.1b	13.2 ± 5.1a	0.16 ± 0.09a	150.5 ± 11.8a
Region	48	22.6 ± 9.1	11.7 ± 6.0	59.7 ± 25.8	80.5 ± 13.2	9.0 ± 3.9	14.6 ± 6.2	0.17 ± 0.07	156.3 ± 35.0
Analysis of variance (*p* value)									
Slope position		< 0.01**	0.680^NS^	0.232^NS^	0.110^NS^	< 0.001***	0.900^NS^	0.834^NS^	0.075^NS^
Year		0.147^NS^	0.351^NS^	0.265^NS^	0.140^NS^	0.622^NS^	0.269^NS^	0.276^NS^	0.056^NS^
Slope position × Year		0.853^NS^	0.984^NS^	0.954^NS^	0.939^NS^	0.919^NS^	0.995^NS^	0.992^NS^	0.932^NS^

CCI, leaf chlorophyll content index; LN, leaf number; SL, shoot length; SLA, single leaf area; SDW, shoot dry weight; LDW, leaf dry weight; SSL, shoot dry weight per unit shoot length; and LLA, leaf dry weight of shoot per unit leaf area. **p < 0.01, ***p < 0.001.; NS, no significant difference.

### 3.4 Effects of slope position on yield of kiwifruit

At harvest, FN, FW, yield, and WP_c_ were 63.9−85.5, 62.9−64.3 g, 6.16−8.58 t ha^−1^, and 1.060−1.429 kg m^−3^, respectively (**Table 5**). Slope position significantly influenced yield components and WP_c_ (*p* < 0.05). Compared with lower slopes, upper slopes increased FN by 33.8%, yield by 39.3%, and WP_c_ by 34.8%. A slightly higher FW was also observed on upper slopes (*p* > 0.05), which was 2.2% higher than that on lower slopes. These results demonstrate that the position of upper slopes can promote kiwifruit yield formation in the subtropical humid region.

**Table 5:** Kiwifruit yield and its components at harvest.

Slope position	*n*	FN	FW (g)	Yield (t ha^−1^)	WP_c_ (kg m^−3^)
Upper	24	85.5 ± 14.8a	64.3 ± 10.1a	8.58 ± 2.36a	1.429 ± 0.29a
Lower	24	63.9 ± 17.8b	62.9 ± 10.5a	6.16 ± 1.56b	1.060 ± 0.20b
Region	48	76.1 ± 16.2	63.7 ± 10.3	7.57 ± 2.39	1.267 ± 0.31
Analysis of variance (*p* value)					
Slope position		< 0.01**	0.484^NS^	< 0.001***	< 0.001***
Year		0.057^NS^	0.211^NS^	0.101^NS^	0.070^NS^
Slope position × Year		0.934^NS^	0.989^NS^	0.849^NS^	0.852^NS^

FN is fruit number per vine; FW is individual fruit weight; and WP_c_ is water productivity. Different lowercase letters with a column denote significant differences between upper and lower slopes at *α* = 0.05. **p < 0.01; ***p < 0.001; NS, no significant difference.

### 3.5 Selection of potential factors influencing the kiwifruit yield under sloping cultivation

**Figure 5:**
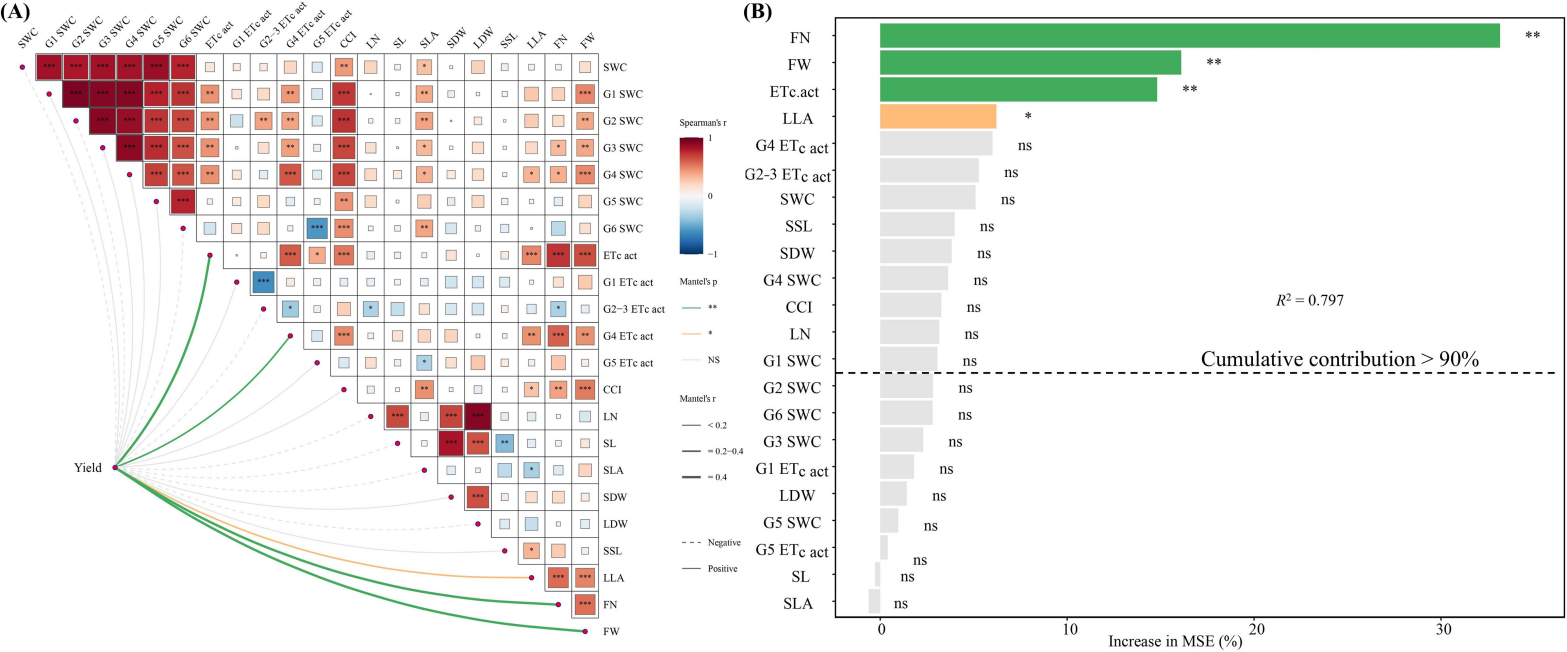
Mantel test analysis between yield and soil water, ET_c act_, and growth physiology **(A)**, and random forest analysis demonstrating the potential important factors of soil water, water consumption, growth physiology, and yield component for yield **(B)**. SWC is the average soil water content in the main root zone during the growing season. G1-G6 correspond to bud-leaf development, flowering, fruit setting, fruit development, fruit maturity, and harvest stages, respectively. CCI, leaf chlorophyll content index; LN, leaf number; SL, shoot length; SLA, single leaf area; SDW, shoot dry weight; LDW, leaf dry weight; SSL, shoot dry weight per unit shoot length; LLA, leaf dry weight of shoot per unit leaf area; FN, fruit number; and FW, fruit weight. *p < 0.05; **p < 0.01, and ***p < 0.001.

Yield was significantly positively correlated with ET_c act_, ET_c act_ at G4, LLA, FN, and FW (Mantel’s *r* = 0.220−0.845 and Mantel’s *p* < 0.05, **Figure 5A**). Moreover, Spearman correlation analysis demonstrated that there were significantly positive correlations among CCI and most variables of soil water and water consumption (*r* > 0.452). RF analysis identified 13 potential variables related to soil water (average SWC, SWC at G1, and SWC at G4), water consumption (ET_c act_, ET_c act_ at G2−3, and ET_c act_ at G4), growth physiology (LLA, SSL, SDW, CCI, and LN), and yield component (FN and FW) can affect yield (*R*^2^ = 79.7%, **Figure 5B**). Specially, FN was determined as the most important factor, with an increase in MSE of 33.2%.

### 3.6 Influence pathway for yield of kiwifruit under sloping cultivation

**Figure 6:**
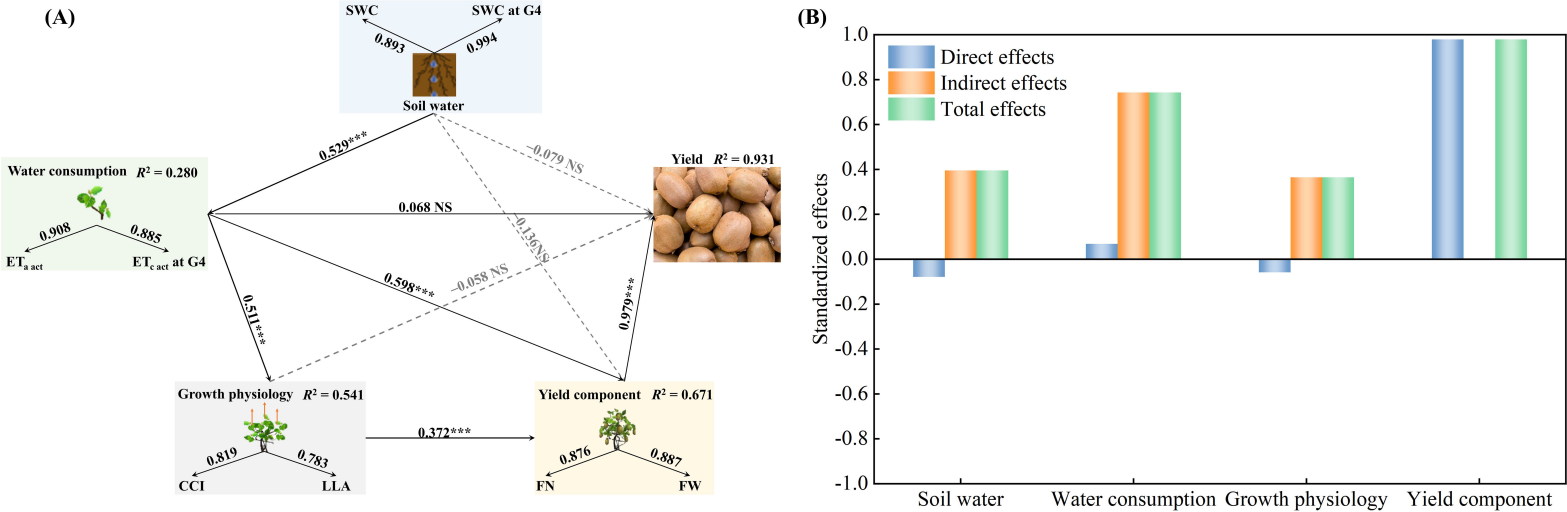
Direct and indirect effects on yield using PLS-PM **(A)**, and standardized direct and indirect effects on yield **(B)**. SWC is the average soil water content in the main root zone during the growing season. SWC at G4 is the soil water content in the main root zone at fruit development stage. ET_c act_ at G4 is the actual evapotranspiration at fruit development stage. CCI, leaf chlorophyll content index; LLA, leaf dry weight of shoot per unit leaf area; FN, fruit number; and FW, fruit weight. ***p < 0.001; NS, not significant.

After screening suitable factors using collinearity diagnostics (VIF < 5) and retaining variables with the highest importance from RF analysis (cumulative contribution > 90%), PLS-PM was adopted to establish the direct and indirect pathways influencing yield. The explanatory variables jointly explained 93.1% of the yield variation (GoF = 0.687, **Figure 6A**). Yield components (FN and FW) had a significantly positive direct effect on yield (0.979, *p* < 0.05). Soil water (average SWC and SWC at G4) and water consumption (ET_c act_ and ET_c act_ at G4) indirectly and positively influenced yield by affecting growth physiology (CCI and LLA) and yield components (path coefficients: 0.395 and 0.742, respectively, *p* < 0.05, **Figure 6B**). Furthermore, growth physiology also indirectly influenced yield through its effects on yield components.

## 4 Discussion

### 4.1 Effects of slope position on soil water and ET_c act_ of kiwifruit during growing season

In the present study, slope position significantly affected the spatial and temporal variations in soil water. Generally, SWC on both slopes increased with depth in the 0−80 cm soil layer (**Figure 3**). Surface soil was influenced by soil evaporation, precipitation, and root water uptake, whereas fewer roots were concentrated in deeper layers, leading to weaker root water uptake (Zhao et al., 2025). As a result, SWC exhibited a gradual increase with increasing soil depth. Moreover, the lower CV of SWC below 80 cm depth and no significant difference in SWC between the upper and lower sections of the 60−80 cm soil layer (**Figure 3**) indicate that kiwifruit roots were mainly distributed in the 0−80 cm soil layer. This finding is consistent with previous studies on kiwifruit (Green and Clothier, 1995; Wang et al., 2010). Specially, Wang et al. (2010) noted that 72.75% of the lateral roots of kiwifruit were distributed in the 0−60 cm soil layer, while 92.07% of fine roots were distributed in the 0−80 cm soil layer, with a significant reduction in root density beyond 80 cm in the horizontal direction. Consequently, the 80 cm depth can be considered the main root water exchange zone or effective root zone for kiwifruit.

As the growing season progressed, SWC on both slopes initially increased and then declined, reaching a maximum at G5. This peak is attributed to high and concentrated precipitation during this period (**Figure 2**), as SWC is generally positively correlated with precipitation (Koster et al., 2004). After the rainfall, soil water was redistributed through root water uptake, evaporation, and gravity drainage (Shan et al., 2023), causing fluctuations in SWC within the main root zone and a subsequent reduction in SWC at harvest (**Figure 4**). Additionally, SWC on upper slopes was significantly lower than that on lower slopes during the growing season (*p* < 0.05, **Figures 3**, **4**), consistent with the findings of Shan et al. (2023) in the loess gully region. On upper slopes, gravity can facilitate downward water infiltration through macropores, and lateral flow along the slope can contribute to the water replenishment lower slopes, thereby reducing water retention in the soils of upper slopes (Lee and Kim, 2019).

Slope position also significantly influenced ET_c act_ of kiwifruit. Low variability in ET_c act_ (CV < 0.1, **Table 2**) was found during the growing season, indicating stable water consumption influenced by precipitation, soil water storage, and soil water exchange. Furthermore, ET_c act_ was significantly higher on upper slopes than on lower slopes (*p* < 0.05, **Table 2**). This finding is in agreement with the findings of Bosquilia et al. (2019), who reported an increase in ET_c act_ at higher slope positions. This phenomenon can be attributed to differences in plant transpiration and soil evaporation. Upper slopes may experience higher soil evaporation due to greater solar radiation, longer sunshine duration, and higher wind speeds compared to lower slopes (Lin et al., 2023). Simultaneously, these conditions may also enhance photosynthesis and leaf transpiration rate (Bansouleh et al., 2009) resulted from higher CCI (**Table 4**), thereby contributing to a larger ET_c act_. Notably, higher ET_c act_ was observed at G4. One reason is higher air temperature and radiation from July to August at the experimental site (**Figure 1**). Another reason is the higher water demand for canopy transpiration and fruit growth associated with the vigorous growth of kiwifruit vines (He et al., 2023).

### 4.2 Effects of slope position on growth physiology of kiwifruit

Slope position reflects the gradient variation in ecological factors, such as water availability, nutrient levels, and sunlight exposure, which directly or indirectly affect plant growth and physiological processes. In this study, global Spearman correlation analysis revealed that a significant positive correlation between CCI and SWC (**Figure 5**). However, significantly lower CCI was observed on lower slopes where significantly higher SWC was detected (**Figure 3**; **Table 4**). The apparent contradiction indicates that chlorophyll content can be not only related to soil water, but may also related to other environmental factors, such as temperature, light, and soil nutrient (Chen et al., 2023; Koide and Kobayashi, 2025) in this subtropical humid region. Due to poorer soil drainage associated with silty clay loam with 12.3% sand content on lower slopes during rainy season in the subtropical humid region, SWC in the main root zone ranged from 80.1%−88.4% FC throughout the growing season, substantially exceeding the commonly recommended SWC threshold for kiwifruit growth (60%−80% FC, **Figure 4**) (He et al., 2023). Xia et al. (2026) also noted that lower sand content (< 12.85%) inhibits water infiltration, leading to water accumulation. Prolonged high SWC in the clay loam can reduce air-filled porosity below critical levels for adequate oxygen diffusion (Dorau et al., 2018), thereby creating conditions of insufficient soil aeration in the root zone. In some fine-textured soils, aeration is a limiting factor for plant growth even at SWC near FC (Ahmad and Li, 2021), particularly under excess water or in the absence of a proper drainage system (Morales-Olmedo et al., 2015). Uteau et al. (2015) noted that oxygen transport was limited under near FC up to -200 hPa, causing a decline in oxygen partial pressures. Likely, Balogh et al. (2011) found higher respiration rates under SWC around 30%−40% in a structured silty clay loam. In contrast, upper slopes had lower SWC (72.5%−87.4% FC, **Figure 4**), which remained within or closer to the recommended range for most of the growing season, avoiding the detrimental effects of excessive soil water. Therefore, the relatively lower SWC on upper slopes may thus have favored better root function and nutrient acquisition, supporting higher chlorophyll synthesis. Additionally, upper slopes generally receive more direct solar radiation, which can alter canopy architecture. The middle canopy, where CCI measured, may experience moderate shading by upper leaves, stimulating the synthesize of photosynthetic pigments to improve light capture (Deng et al., 2023). Consistent with these advantages, upper slopes exhibited significantly higher SDW relative to lower slopes (*p* < 0.05, **Table 5**), aligning with the findings of Sun et al. (2021) on citrus, which attributed this to stronger sunlight and higher effective radiation on upper slopes, promoting dry matter accumulation (Lee et al., 2023).

### 4.3 Main factors influencing the kiwifruit yield

Yield is an important economic indicator for evaluating the sustainable development of kiwifruit production (Wang et al., 2024). Under sloping cultivation, kiwifruit yield formation was collectively influenced by soil water, water consumption, growth physiology, and yield components. Yield formation was primarily driven by yield components (FN and FW), which exerted the strongest direct positive effect on yield (direct path coefficient = 0.979, *p* < 0.05, **Figure 6**). This finding is consistent with previous studies that noted that the dominant role of FN and FW in final yield determination in fruit crops (Richardson and McAneney, 1990; Marini, 2003). Notably, FN was significantly higher on upper slopes than on lower slopes (*p* < 0.05, **Table 5**), and FW was slightly higher on upper slopes, jointly contributing to the higher yield observed on upper slopes.

Beyond direct effects, soil water (average SWC and SWC at G4) and water consumption (ET_c act_ and ET_c act_ at G4) modulated growth physiology (CCI and LLA), which influenced yield components, thereby indirectly affecting yield (*p* < 0.05, **Figure 6**). In this indirect pathway, CCI and LLA were identified as key intermediate growth physiology variables. CCI reflects leaf photosynthetic capacity and nitrogen status (Immanuel and Miruna, 2024), while LLA can indirectly indicate the efficiency of leaf dry matter investment and canopy structure (Sullivan et al., 2026). Both soil water and water consumption exerted significant positive effects on growth physiology (**Figure 6**). Consistent with topographic hydrological patterns, gravity-driven lateral flow transports soil water and associated soluble nutrients (including nitrate nitrogen and potassium) from upper to lower slopes (Wezel et al., 2002; Shan et al., 2023), theoretically creating higher nutrient availability at lower slope positions. However, our observation of significantly higher CCI and LLA on upper slopes (**Table 4**) is inconsistent with this expectation, revealing previously unrecognized constraints on nutrient utilization efficiency on lower slopes. On upper slopes, SWC remained within a range conductive to kiwifruit growth (72.5%-87.4% FC, **Figure 4**), while ET_c act_ was significantly higher than on lower slopes (**Table 2**). This higher ET_c act_ further reflects enhanced transpiration and photosynthetic activity (Jiang et al., 2022), which promoted dry matter accumulation and fruit set (Bosquilia et al., 2019). In contrast, lower slopes in this subtropical humid region frequently experienced SWC exceeding the optimal upper threshold (80.1%-88.4% FC, **Figure 4**), leading to potential excessive water stress, particularly at G4 when concentrated and high-intensity precipitation occurred. Due to the sensitivity of kiwifruit to soil water (Calabritto et al., 2024; Zheng et al., 2024), this excessive water likely impaired root function and reduced active water and nutrient uptake (Rajan et al., 2024; Ding et al., 2025). Although direct measurements of root hypoxia (e.g. root alcohol dehydrogenase activity or soil oxygen concentration) were not conducted in this study, Ding et al. (2025) demonstrated that prolonged excessive SWC in heavy soils significantly reduced root hydraulic conductance and nutrient adsorption in kiwifruit. These impairments likely constrained CCI and LLA on lower slopes, and may contribute to lower ET_c act_ and nutrient utilization. Notably, these findings differed from our initial hypothesis that moderate water deficit on upper slopes might directly enhance yield. Instead, the relatively lower SWC on upper slopes fell within the appropriate range (60%-80% FC) across most growth stages, thereby avoiding the negative effects of excessive soil water accumulation and supporting superior growth physiology and yield formation compared to lower slopes.

### 4.4 Management implications for sloping cultivation in a subtropical humid region

Our findings demonstrate that compared to lower slopes, upper slopes provided more suitable soil water conditions (72.5%–87.4% FC) for kiwifruit growth, leading to higher yield and WP_c_. In contrast, lower slopes frequently experienced excessive soil water (80.1%–88.4% FC) that exceeded the optimal threshold (80% FC), potentially impairing root function, chlorophyll synthesis, and dry matter accumulation. Therefore, for sloping kiwifruit orchards in subtropical humid regions, upper or mid−upper slopes for new plantings are recommended to avoid excessive water in soil. On lower slopes, drainage improvements such as deeper open ditches or raised beds should be implemented to reduce SWC during rainy seasons (Qi et al., 2025), especially at G4. Although upper slopes had relatively lower SWC, the ET_c act_ was significantly higher, indicating active transpiration. As a result, supplemental irrigation on upper slopes during dry periods could potentially stabilize yield (Rey et al., 2016). Additionally, on upper slopes, moderate pruning should be maintained to optimize light distribution and enhance CCI and LLA, while on lower slopes, excessive nutrient application should be avoided and soil aeration improved to mitigate root hypoxia.

## 5 Conclusions

Based on a field investigation, this study systematically evaluated the effects of slope position on soil water dynamics and kiwifruit yield in a subtropical humid region. The differences in spatiotemporal soil water distribution, ET_c act_, growth physiological indicators, and yield between upper and lower slopes were analyzed. Subsequently, the key factors influencing yield were identified under sloping cultivation. The results revealed that slope position significantly influenced the spatial and temporal distribution of soil water during the growing season (*p* < 0.01). Specifically, SWC in the main root zone was significantly lower on upper slopes than on lower slopes (*p* < 0.05), with more appropriate soil water conditions for kiwifruit growth. Superior growth physiology, WP_c_, and yield performance were observed on upper slopes. PLS-PM identified yield components (FN and FW) as direct factors influencing yield. Soil water (average SWC and SWC at G4) and water consumption (ET_c act_ and ET_c act_ at G4) indirectly and positively affected yield, by influencing growth physiology (LLA and CCI) and yield components. This study can provide guidance for optimizing sloping land cultivation to promote sustainable kiwifruit production. In practice, supplemental irrigation on upper slopes during dry seasons is recommended to stabilize yield, whereas drainage improvements on lower slopes are critical to prevent excessive soil water, particularly during the fruit development stage. However, several limitations should be noted that no measurements of direct nutrient status and physiological processes limited a mechanistic interpretation of hypoxia on lower slopes. Therefore, the future studies are required to elucidate the underlying mechanism.

## Data Availability

The raw data supporting the conclusions of this article will be made available by the authors, without undue reservation.

## References

[B1] Ahmad H. Li J. (2021). Impact of water deficit on the development and senescence of tomato roots grown under various soil textures of Shaanxi, China. BMC Plant Biol. 21, 241. doi: 10.1186/s12870-021-03018-1. PMID: 34049491 PMC8162013

[B2] Allen R. G. Pereira L. S. Raes D. Smith M. (1998). Crop evapotranspiration-Guidelines for computing crop water requirements-FAO Irrigation and drainage paper 56 Vol. 300 (Rome: FAO), D05109.

[B3] Balogh J. Pintér K. Fóti S. Cserhalmi D. Papp M. Nagy Z. (2011). Dependence of soil respiration on soil moisture, clay content, soil organic matter, and CO2 uptake in dry grasslands. Soil Biol. Biochem. 43, 1006–1013. doi: 10.1016/j.soilbio.2011.01.017

[B4] Bansouleh B. F. Sharifi M. Van Keulen H. (2009). Sensitivity analysis of performance of crop growth simulation models to daily solar radiation estimation methods in Iran. Energy Convers. Manage. 50, 2826–2836. doi: 10.1016/j.enconman.2009.06.028. PMID: 38826717

[B5] Bosquilia R. W. Neale C. M. Duarte S. N. Longhi S. J. Ferraz S. F. D. B. Muller-Karger F. E. . (2019). Evaluation of evapotranspiration variations as a function of relief and terrain exposure through multivariate statistical analysis. Ecohydrol. Hydrobiol. 19, 307–315. doi: 10.1016/j.ecohyd.2018.11.001. PMID: 38826717

[B6] Calabritto M. Mininni A. N. Di Biase R. Pietrafesa A. Dichio B. (2024). Spatio-temporal dynamics of root water uptake and identification of soil moisture thresholds for precision irrigation in a Mediterranean yellow-fleshed kiwifruit orchard. Front. Plant Sci. 15, 1472093. doi: 10.3389/fpls.2024.1472093. PMID: 39554527 PMC11563807

[B7] Chen K. Pan Y. Li Y. Cheng J. Lin H. Zhuo W. . (2023). Slope position-mediated soil environmental filtering drives plant community assembly processes in hilly shrublands of Guilin, China. Front. Plant Sci. 13, 1074191. doi: 10.3389/fpls.2022.1074191. PMID: 36684746 PMC9859686

[B8] Chen C. Zou X. Singh A. K. Zhu X. Zhang W. Yang B. . (2021). Effects of hillslope position on soil water infiltration and preferential flow in tropical forest in southwest China. J. Environ. Manage. 299, 113672. doi: 10.1016/j.jenvman.2021.113672. PMID: 34488112

[B9] Deng H. Li Y. Pang C. Zhang K. Tian X. Wang T. . (2023). Significant increases in Donghong kiwifruit yield by a novel umbrella-shaped trellis system and identification of associated molecular mechanisms. Front. Plant Sci. 14, 1143525. doi: 10.3389/fpls.2023.1143525. PMID: 36993843 PMC10040675

[B10] Ding Y. Shang C. Zhao L. Jin S. Li C. Yin S. . (2025). Effects of irrigation and fertilization management on kiwifruit yield, water use efficiency and quality in China: A meta-analysis. Front. Plant Sci. 16, 1534702. doi: 10.3389/fpls.2025.1534702. PMID: 41169724 PMC12568633

[B11] Dorau K. Luster J. Mansfeldt T. (2018). Soil aeration: the relation between air-filled pore volume and redox potential. Eur. J. Soil Sci. 69, 1035–1043. doi: 10.1111/ejss.12717. PMID: 40046247

[B12] Fernández J. E. Alcon F. Diaz-Espejo A. Hernandez-Santana V. Cuevas M. (2020). Water use indicators and economic analysis for on-farm irrigation decision: A case study of a super high density olive tree orchard. Agric. Water Manage. 237, 106074. doi: 10.1016/j.agwat.2020.106074. PMID: 38826717

[B13] Gao R. Guan Y. He X. Wang J. Fan D. Ma Y. . (2026). Multi-factor driving force analysis of soil salinization in desert–oasis regions using satellite data. Water 18, 133. doi: 10.3390/w18010133. PMID: 30654563

[B14] Ghimire K. McIntyre I. Caffe M. (2024). Evaluation of morpho-physiological traits of oat (Avena sativa L.) under drought stress. Agriculture 14, 109. doi: 10.3390/agriculture14010109. PMID: 30654563

[B15] Green S. R. Clothier B. E. (1995). Root water uptake by kiwifruit vines following partial wetting of the root zone. Plant Soil 173, 317–328. doi: 10.1007/BF00011470. PMID: 30311153

[B16] He Z. Lu X. Cui N. Jiang S. Zheng S. Chen F. . (2023). Effect of soil water content threshold on kiwifruit quality at different growth stages with drip irrigation in the humid area of Southern China. Sci. Hortic. 307, 111477. doi: 10.1016/j.scienta.2022.111477. PMID: 38826717

[B17] Heslop A. D. Jahufer Z. Hofmann R. W. (2023). Responses to water stress extremes in diverse red clover germplasm accessions. Front. Plant Sci. 14, 1195058. doi: 10.3389/fpls.2023.1195058. PMID: 37426971 PMC10325626

[B18] Horel Á. Zsigmond T. (2023). Plant growth and soil water content changes under different inter-row soil management methods in a sloping vineyard. Plants 12, 1549. doi: 10.3390/plants12071549. PMID: 37050175 PMC10096666

[B19] Immanuel R. Miruna M. (2024). Quantifying chlorophyll content index for efficient nitrogen management in rice (Oryza sativa L.). Crop Res.(0970-4884) 59, 196. doi: 10.31830/2454-1761.2024.CR-981

[B20] Jiang S. Liang C. Zhao L. Gong D. Huang Y. Xing L. . (2022). Energy and evapotranspiration partitioning over a humid region orchard: Field measurements and partitioning model comparisons. J. Hydrol. 610, 127890. doi: 10.1016/j.jhydrol.2022.127890. PMID: 38826717

[B21] Koide D. Kobayashi M. (2025). Chlorophyll and topographic patterns demonstrate stress conditions drive the brightness of autumn leaf colour. Plant Biol. 27, 279–286. doi: 10.1111/plb.13755. PMID: 39720946

[B22] Koster R. D. Dirmeyer P. A. Guo Z. Bonan G. Chan E. Cox P. . (2004). Regions of strong coupling between soil moisture and precipitation. Science 305, 1138–1140. doi: 10.1126/science.1100217. PMID: 15326351

[B23] Lee E. Kim S. (2019). Seasonal and spatial characterization of soil moisture and soil water tension in a steep hillslope. J. Hydrol. 568, 676–685. doi: 10.1016/j.jhydrol.2018.11.027. PMID: 38826717

[B24] Lee S. Yeo H. J. Lee S. Y. Kim S. R. Park S. U. Park C. H. (2023). The effect of light and dark treatment on the production of rosmarinic acid and biological activities in Perilla frutescens microgreens. Plants 12, 1613. doi: 10.3390/plants12081613. PMID: 37111837 PMC10142874

[B25] Li Y. Sun J. Liu J. Yuan Z. Hu S. Li X. . (2025). Straw returning to the field alleviates drought stress in maize by enhancing radiation use efficiency to promote agronomic character and dry matter accumulation. J. Agric. Food Res. 23, 102243. doi: 10.1016/j.jafr.2025.102243. PMID: 38826717

[B26] Lin D. Katurji M. Revell L. E. Khan B. Sturman A. (2023). Investigating multiscale meteorological controls and impact of soil moisture heterogeneity on radiation fog in complex terrain using semi-idealised simulations. Atmos. Chem. Phys. 23, 14451–14479. doi: 10.5194/acp-23-14451-2023

[B27] Marini R. P. (2003). Peach fruit weight, yield, and crop value are affected by number of fruiting shoots per tree. HortScience 38, 512–514. doi: 10.21273/HORTSCI.38.4.512

[B28] Monsieurs E. Dessie M. Adgo E. Poesen J. Deckers J. Verhoest N. . (2015). Seasonal surface drainage of sloping farmland: a review of its hydrogeomorphic impacts. Land Degrad. Dev. 26, 35–44. doi: 10.1002/ldr.2286. PMID: 41531421

[B29] Morales-Olmedo M. Ortiz M. Sellés G. (2015). Effects of transient soil waterlogging and its importance for rootstock selection. Chil. J. Agric. Res. 75, 45–56. doi: 10.4067/S0718-58392015000300006

[B30] Mualem Y. (1976). A new model for predicting the hydraulic conductivity of unsaturated porous media. Water Resour. Res. 12, 513–522. doi: 10.1029/WR012i003p00513. PMID: 29143083

[B31] Niu Z. Wang J. Shi L. Yao L. Zhang Y. Si H. . (2026). Construction of a soil organic matter estimation model based on continuous wavelet transform combined with different modelling methods. Plant Soil 520, 1037-1052. doi: 10.1007/s11104-026-08309-w. PMID: 30311153

[B32] Padilla F. M. Gallardo M. Peña-Fleitas M. T. De Souza R. Thompson R. B. (2018). Proximal optical sensors for nitrogen management of vegetable crops: A review. Sensors 18, 2083. doi: 10.3390/s18072083. PMID: 29958482 PMC6069161

[B33] Parry C. Blonquist J. J. Bugbee B. (2014). In situ measurement of leaf chlorophyll concentration: analysis of the optical/absolute relationship. Plant Cell Environ. 37, 2508–2520. doi: 10.1111/pce.12324. PMID: 24635697

[B34] Phi S. Clarke W. Li L. (2013). Laboratory and numerical investigations of hillslope soil saturation development and runoff generation over rainfall events. J. Hydrol. 493, 1–15. doi: 10.1016/j.jhydrol.2013.04.009. PMID: 38826717

[B35] Qi B. Yang S. Li D. Qin D. Zheng X. Hu J. . (2025). Effects of drainage technology on waterlogging reduction and rice yield in mid-lower reaches of Yangtze River. Agronomy 15, 905. doi: 10.3390/agronomy15040905. PMID: 30654563

[B36] Rajan P. Natraj P. Kim M. Lee M. Jang Y. J. Lee Y. J. . (2024). Climate change impacts on and response strategies for kiwifruit production: A comprehensive review. Plants 13, 2354. doi: 10.3390/plants13172354. PMID: 39273838 PMC11396826

[B37] Rana V. S. Zarea S. E. Sharma S. Rana N. Kumar V. Sharma U. (2023). Differential response of the leaf fruit ratio and girdling on the leaf nutrient concentrations, yield, and quality of nectarine. J. Plant Growth Regul. 42, 2360–2373. doi: 10.1007/s00344-022-10710-5. PMID: 30311153

[B38] Rey D. Holman I. Daccache A. Morris J. Weatherhead E. Knox J. (2016). Modelling and mapping the economic value of supplemental irrigation in a humid climate. Agric. Water Manage. 173, 13–22. doi: 10.1016/j.agwat.2016.04.017. PMID: 38826717

[B39] Richardson A. C. McAneney K. J. (1990). Influence of fruit number on fruit weight and yield of kiwifruit. Sci. Hortic. 42, 233–241. doi: 10.1016/0304-4238(90)90085-S

[B40] Rowland S. D. Zumstein K. Nakayama H. Cheng Z. Flores A. M. Chitwood D. H. . (2020). Leaf shape is a predictor of fruit quality and cultivar performance in tomato. New Phytol. 226, 851–865. doi: 10.1111/nph.16403. PMID: 31880321 PMC7187315

[B41] Shan Y. Xie J. Lei N. (2023). Spatial distribution characteristics of soil moisture in different slopes on Loess Gully Region. Agron. J. 115, 997–1005. doi: 10.1002/agj2.21226. PMID: 41531421

[B42] Sullivan F. B. Hastings J. H. Ollinger S. V. Ouimette A. Richardson A. D. Palace M. (2026). Parsing the relative contributions of leaf and canopy traits in airborne spectrometer measurements. Remote Sens. 18, 355. doi: 10.3390/rs18020355. PMID: 30654563

[B43] Sun S. Zhang G. He T. Song S. Chu X. (2021). Effects of landscape positions and landscape types on soil properties and chlorophyll content of citrus in a sloping orchard in the Three Gorges Reservoir area, China. Sustainability 13, 4288. doi: 10.3390/su13084288. PMID: 30654563

[B44] Tan S. Wang Q. Zhang J. Chen Y. Shan Y. Xu D. (2018). Performance of AquaCrop model for cotton growth simulation under film-mulched drip irrigation in southern Xinjiang, China. Agric. Water Manage. 196, 99–113. doi: 10.1016/j.agwat.2017.11.001. PMID: 38826717

[B45] Tang W. Xiao X. Tang H. Yang G. (2014). Effects of different planting patterns on water use of soil and crops annual productivity in southern hilly dryland. Sci. Agric. Sin. 47, 3606–3617. doi: 10.3864/j.issn.0578-1752.2014.18.009. PMID: 41470978

[B46] Uteau D. Hafner S. Pagenkemper S. K. Peth S. Wiesenberg G. L. Kuzyakov Y. . (2015). Oxygen and redox potential gradients in the rhizosphere of alfalfa grown on a loamy soil. J. Plant Nutr. Soil Sci. 178, 278–287. doi: 10.1002/jpln.201300624

[B47] van Genuchten M. T. (1980). A closed-form equation for predicting the hydraulic conductivity of unsaturated soils. Soil Sci. Soc Am. J. 44, 892–898. doi: 10.2136/sssaj1980.03615995004400050002x

[B48] Wang J. Tong Y. Gao Y. (2010). Study on the roots distribution and growth dynamics of kiwifruit in northern area of Qingling. J. Anhui Agric. Sci. 38, 8085–8087.

[B49] Wang Y. Wu Y. Wang X. Ren W. Chen Q. Zhang S. . (2024). Genome wide association analysis identifies candidate genes for fruit quality and yield in Actinidia eriantha. J. Integr. Agric. 23, 1929–1939. doi: 10.1016/j.jia.2023.11.025. PMID: 38826717

[B50] Wezel A. Steinmüller N. Friederichsen J. R. (2002). Slope position effects on soil fertility and crop productivity and implications for soil conservation in upland northwest Vietnam. Agric. Ecosyst. Environ. 91, 113–126. doi: 10.1016/S0167-8809(01)00242-0

[B51] Xia C. Ren C. Wang Y. Wang Z. Jia M. Xi Y. . (2026). Decoding soil-topography buffering of maize yield spatial heterogeneity in extreme precipitation year using Sentinel-2 data and SHAP interpretability. Field Crops Res. 337, 110263. doi: 10.1016/j.fcr.2025.110263. PMID: 38826717

[B52] Xue J. Fan Y. Su B. Fuentes S. (2019). Assessment of canopy vigor information from kiwifruit plants based on a digital surface model from unmanned aerial vehicle imagery. Int. J. Agric. Biol. Eng. 12, 165–171. doi: 10.25165/j.ijabe.20191201.4634

[B53] Yao Z. Zhang Y. Yang Q. Zhang L. Wang L. Zhao D. (2024). Simulation of topographic effects on soil erosion and deposition in a small watershed of loess hilly region. Earth Surf. Process. Landf. 49, 1836–1848. doi: 10.1002/esp.5801. PMID: 41531421

[B54] Yetik A. K. Candoğan B. N. (2023). Chlorophyll response to water stress and the potential of using crop water stress index in sugar beet farming. Sugar Tech. 25, 57–68. doi: 10.1007/s12355-022-01184-6. PMID: 35966232 PMC9362222

[B55] Zhang P. Guo Y. Yao Y. Qi W. Teng J. (2023). Root distribution characteristics of Caragana korshinskii and their relationship with soil water content in different slope positions. J. Northwest. A&F Univ. (Nat. Sci. Ed.) 51, 28–39.

[B56] Zhang X. Zhao W. Wang L. Liu Y. Liu Y. Feng Q. (2019). Relationship between soil water content and soil particle size on typical slopes of the Loess Plateau during a drought year. Sci. Total Environ. 648, 943–954. doi: 10.1016/j.scitotenv.2018.08.211. PMID: 30144762

[B57] Zhao Z. Tang G. Li J. Wu M. Zhang L. (2025). Water source of artificial plants in the northeastern margin of Tengger Desert based on hydrogen and oxygen stable isotopes. Front. Plant Sci. 16, 1523085. doi: 10.3389/fpls.2025.1523085. PMID: 40391036 PMC12086160

[B58] Zheng S. Jiang S. Cui N. Zhao L. Gong D. Wang Y. . (2023). Deficit drip irrigation improves kiwifruit quality and water productivity under rain-shelter cultivation in the humid area of South China. Agric. Water Manage. 289, 108530. doi: 10.1016/j.agwat.2023.108530. PMID: 38826717

[B59] Zheng W. Wang N. Qian G. Qian X. Liu W. Huang L. (2024). Cross-niche protection of kiwi plant against above-ground canker disease by beneficial rhizosphere Flavobacterium. Commun. Biol. 7, 1458. doi: 10.1038/s42003-024-07208-z. PMID: 39511396 PMC11543660

[B60] Zhu Q. Schmidt J. P. Bryant R. B. (2015). Maize (Zea mays L.) yield response to nitrogen as influenced by spatio-temporal variations of soil–water-topography dynamics. Soil Tillage Res. 146, 174–183. doi: 10.1016/j.still.2014.10.006. PMID: 38826717

